# TRIM14 restricts tembusu virus infection through degrading viral NS1 protein and activating type I interferon signaling

**DOI:** 10.1371/journal.ppat.1013200

**Published:** 2025-05-28

**Authors:** Peng Zhou, Qingxiang Zhang, Yueshan Yang, Dan Liu, Wanrong Wu, Anan Jongkaewwattana, Hui Jin, Hongbo Zhou, Rui Luo

**Affiliations:** 1 State Key Laboratory of Agricultural Microbiology, College of Veterinary Medicine, Huazhong Agricultural University, Wuhan, China; 2 The Cooperative Innovation Center for Sustainable Pig Production, Wuhan, China; 3 Key Laboratory of Development of Veterinary Diagnostic Products, Ministry of Agriculture and Rural Affairs of the People’s Republic of China, Wuhan, China; 4 China Institute of Veterinary Drug Control, Beijing, PR China; 5 Virology and Cell Technology Research Team, National Center for Genetic Engineering and Biotechnology (BIOTEC), National Science and Technology Development Agency (NSTDA), Klong Nueng, Pathum Thani, Thailand; University of California Davis, UNITED STATES OF AMERICA

## Abstract

Tembusu virus (TMUV), an emerging avian orthoflavivirus, causes severe economic losses due to egg-drop syndrome and fatal encephalitis in domestic waterfowl. To combat this threat, the host immune system plays a crucial role in controlling and eliminating TMUV infection. Understanding the mechanisms of this immune response is thus vital for developing effective strategies against the virus. In this study, we investigated the antiviral activities of duck TRIM family proteins (duTRIM) against TMUV, focusing particularly on duTRIM14 as a potent host restriction factor. We showed that overexpression of duTRIM14 significantly inhibits TMUV replication, while its deficiency leads to increased viral titers. We elucidate a novel mechanism by which duTRIM14 interacts with the TMUV NS1 protein, facilitating its K27/K29-linked polyubiquitination and subsequent proteasomal degradation. The Lys141 residue on NS1 was identified as critical for this process, with its removal significantly enhancing TMUV replication both *in vitro* and *in vivo*. Furthermore, we showed that duTRIM14 interacts with duck TBK1 (duTBK1), promoting its K63-linked polyubiquitination on Lys30 and Lys401, which substantially augments IFN-β production during TMUV infection. Taken together, these results provide a novel dual-action antiviral mechanism in which duTRIM14 suppresses TMUV replication by simultaneously promoting proteasomal degradation of NS1 and enhancing the host antiviral response by modulating duTBK1 activity.

## Author summary

Tembusu virus (TMUV) is a major pathogen affecting the duck farming industry and causing significant economic losses. This study identifies the duck protein TRIM14 (duTRIM14) as an important host factor that restricts TMUV infection. We show that duTRIM14 employs a dual antiviral mechanism: it directly targets the essential viral protein NS1 and facilitates its degradation within the host cell. At the same time, it enhances the innate immune signaling pathways of the host that are involved in the antiviral defense. The functional significance of the direct interaction with NS1 was confirmed, as altering the target site on NS1 significantly increased viral replication both *in vitro* and *in vivo*. To our knowledge, this is the first report detailing the restriction of TMUV by a host protein through this combined strategy. These results provide important insights into the interactions between host and TMUV and form the basis for the development of improved control strategies against this virus.

## Introduction

Tembusu virus (TMUV), a pathogenic orthoflavivirus belonging to the *Flaviviridae* family, was first isolated from *Culex tritaeniorhynchus* mosquitoes in Malaysia in 1955 [1]. In 2010, TMUV triggered widespread outbreaks in major duck producing regions of China and caused significant economic losses in the duck industry [2]. Infected ducks typically exhibit severe egg-drop syndrome, stunted growth, and neurological symptoms, with morbidity rates as high as 90% and mortality rates around 30% [3,4]. The TMUV replication cycle, like that of other orthoflaviviruses, begins when the virus enters host cells via receptor-mediated endocytosis [5]. The acidic environment of the endosome triggers fusion with the host membrane, releasing the genomic RNA into the cytoplasm [6]. The viral RNA is then translated into a polyprotein, which is subsequently cleaved into three structural proteins (C, prM, and E) and seven non-structural (NS) proteins (NS1, NS2A, NS2B, NS3, NS4A, NS4B, and NS5) [7]. These NS proteins assemble into replication complexes within ER-derived membranous structures, where they facilitate the replication of viral RNA through a negative-strand intermediate, generating new positive-sense RNA genomes [8]. Immature virions are formed by the assembly of newly synthesized viral proteins and RNA within the endoplasmic reticulum (ER) [9]. These particles undergo maturation as they transit through the Golgi apparatus, where host proteases mediate their proteolytic cleavage, resulting in the formation of mature virions [10]. The mature virions are then released from the host cell via exocytosis, allowing the infection of new cells [11]. Among the NS proteins, NS1 plays a multifaceted role in various stages of viral replication [12]. It facilitates the restructuring of cellular membranes to support the formation of the replication complex (RC), which is essential for viral RNA replication [13,14]. Furthermore, NS1 colocalizes with other NS proteins and viral dsRNA within the RC in the ER, underscoring its importance as a cofactor for viral replication [15,16].

The innate immune system serves as the first line of defense against viral invasion. Following TMUV infection, viral RNA in the cytoplasm is rapidly recognized by retinoic acid-inducible gene I (RIG-I)-like receptors (RLRs), including RIG-I and melanoma differentiation-associated gene 5 (MDA5) [17,18]. Upon recognition of viral RNA, RIG-I and MDA5 undergo conformational changes that release their caspase activation and recruitment domains (CARDs) from repression [19,20]. This release enables the CARDs to recruit downstream adaptor proteins, such as mitochondrial antiviral signaling proteins (MAVS), which subsequently leads to the activation of the inhibitor of kappa-B kinase epsilon (IKKε) and TANK-binding kinase 1 (TBK1) [21]. These kinases phosphorylate interferon regulatory factors 3 and 7 (IRF3/7), which triggers their dimerization and nuclear translocation, culminating in the production of type I interferons (IFNs) [22]. The secreted IFNs bind to their receptors and trigger a complex signaling cascade that leads to the expression of hundreds of interferon-stimulated genes (ISGs) and establishes a robust antiviral defense [23].

The Tripartite Motif (TRIM) family of proteins found in most eukaryotes, including birds, comprises at least 80 members characterized by three conserved domains: an N-terminal Really Interesting New Gene (RING) domain, one or two B-Boxes (B1/B2), and a Coiled Coil (CC) domain, collectively referred to as RBCC [24,25]. The RING domains confer the TRIM proteins the ability to function as ubiquitin E3 ligases [26]. These enzymes are crucial for the ubiquitination of target proteins and play a central role in the regulation of innate immunity and the control of antiviral responses [27,28]. For instance, mammalian TRIM35 enhances the RIG-I-mediated antiviral signaling cascade by inducing K63-linked polyubiquitination of TRAF3 and the subsequent formation of a signaling complex with MAVS and TBK1, thereby suppressing Influenza A Virus (IAV) replication [29]. Similarly, chicken TRIM25 restricts Infectious Bursal Disease Virus (IBDV) infection by directly interacting with the VP3 protein, leading to its K27-linked ubiquitination and subsequent proteasomal degradation [30].

In this study, we identified duck TRIM14 (duTRIM14) as a host antiviral protein that counteracts TMUV infection by two distinct mechanisms. First, duTRIM14 interacts with TMUV NS1 and promotes its degradation via K27/K29-linked ubiquitination. Second, duTRIM14 enhances RLR-mediated antiviral immune responses by facilitating K63-linked polyubiquitination of duTBK1, thereby inhibiting TMUV replication. Thus, our research has uncovered a significant mechanism by which duTRIM14 regulates the antiviral response and innate immunity against TMUV infection.

## Results

### DuTRIM14 exhibits potent antiviral activity against TMUV

**Fig 1:**
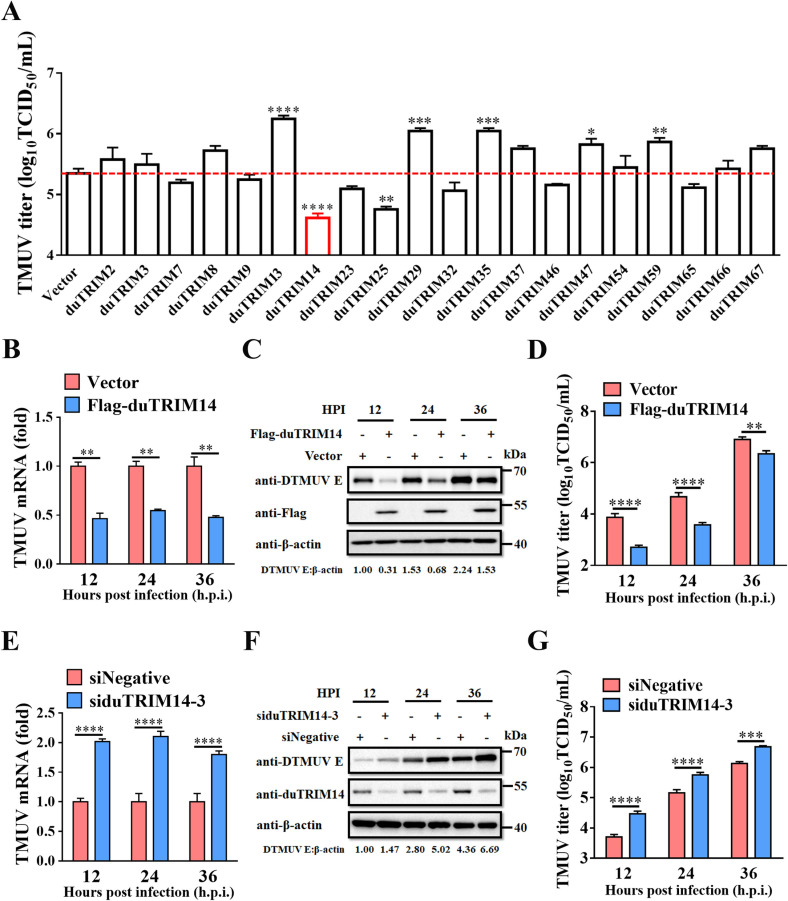
DuTRIM14 exhibits potent antiviral activity against TMUV. (A) DEFs were transfected with plasmids expressing one of twenty duck TRIM proteins, followed by TMUV infection at an MOI of 0.1. Supernatants were collected 24 h post-infection for TCID_50_ assay to measure viral titers. (B-D) DEFs were transfected with plasmids expressing either Flag-duTRIM14 or an empty vector for 24 h, followed by TMUV infection at an MOI of 0.1. Viral mRNA levels (B) and E protein expression (C) were evaluated in cell lysates at indicated time points using RT-qPCR and western blot, respectively. Viral titers (D) in the culture supernatants were measured using TCID_50_ assay. (E-G) DEFs were transfected with either siduTRIM14-3 or a negative control siRNA (siNegative) for 24 h, followed by TMUV infection at an MOI of 0.1. Viral mRNA (E) and E protein expression (F) were evaluated in cell lysates at indicated time points using RT-qPCR and western blot, respectively. Viral titers (G) in the culture supernatants were measured by TCID_50_ assay. All RT-qPCR results are represented as relative fold changes, normalized to GAPDH. Results from RT-qPCR and TCID_50_ assays are presented as means ± SD from three independent experiments. Statistical significance was determined by one-way ANOVA followed by Dunett’s multiple comparisons test (A), or two-way ANOVA followed by Sidak’s multiple comparisons test (B, D, E and G). *, *P* < 0.05; **, *P* < 0.01; ***, *P* < 0.001; ****, *P* < 0.0001.

To systematically evaluate the antiviral effects of duck TRIM (duTRIM) proteins against TMUV, we initially screened 20 duTRIM proteins transiently expressed in duck embryonic fibroblasts (DEFs). Consistent with previous studies [31], we observed that duTRIM25 expression significantly inhibited TMUV replication. Notably, we found that duTRIM14 was the most potent inhibitor of TMUV replication, reducing virus titers by approximately 5.5-fold at 24 h post-infection (h.p.i.) (Fig 1A). Additionally, duTRIM14 mRNA and protein expression were significantly upregulated during TMUV infection (S1A and S1B Fig). To further investigate the antiviral effect of duTRIM14, we transfected DEFs with Flag-duTRIM14 and, 24 h later, infected them with TMUV at a multiplicity of infection (MOI) of 0.1. RT-qPCR analysis revealed a consistent 1.9- to 2.2-fold reduction in viral genome mRNA levels at 12, 24, and 36 h.p.i in duTRIM14-expressing cells compared to controls (Fig 1B). Complementing these findings, western blot assays demonstrated that duTRIM14 overexpression markedly decreased TMUV E protein levels (Fig 1C). Furthermore, TCID_50_ assays confirmed a marked reduction in viral titers in duTRIM14-overexpressing cells (Fig 1D). To further validate these findings, we designed three small interfering RNAs (siRNAs) targeting duTRIM14 mRNA, with siduTRIM14–3 proving most effective in suppressing endogenous duTRIM14 expression, as confirmed by RT-qPCR and western blot (S2A and S2B Fig). Assessment of duTRIM14 knockdown effects on TMUV replication revealed that reducing duTRIM14 levels led to increased viral RNA, TMUV E protein expression, and viral titers, compared to siNegative controls (Fig 1E-1G). Collectively, our findings demonstrated that duTRIM14 is an effective inhibitor of TMUV replication in DEFs.

### DuTRIM14 inhibits TMUV replication by targeting viral NS1 protein

**Fig 2:**
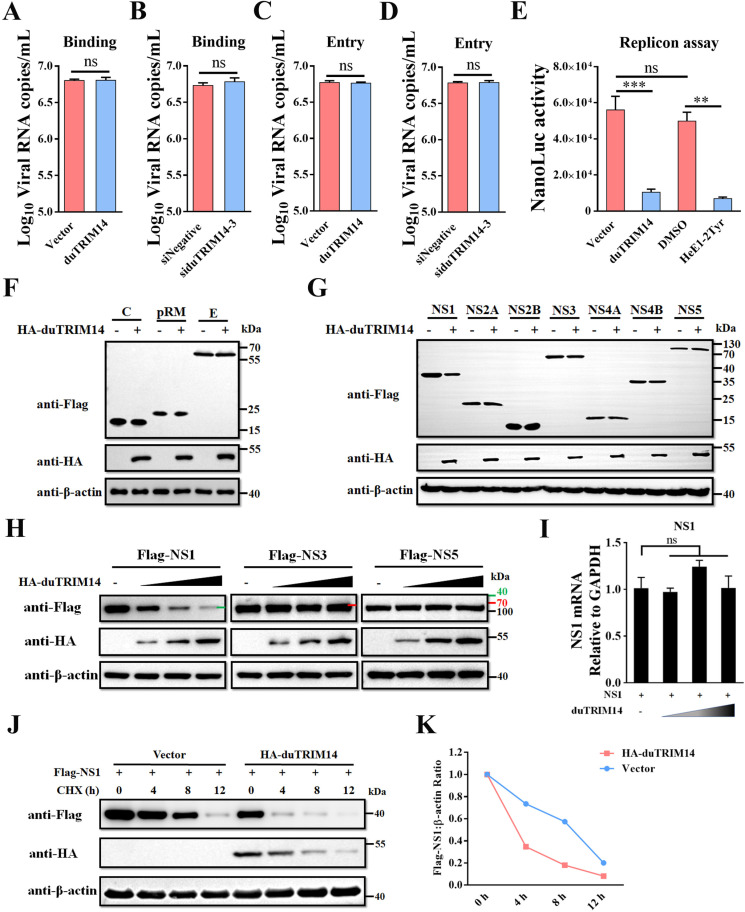
DuTRIM14 inhibits TMUV replication by targeting viral NS1 protein. (A and B) DEFs were transfected with Flag-duTRIM14 (A) or siduTRIM14-3 (B) for 24 h, followed by incubation with TMUV at 4°C for 1 h. The cells were then washed three times with phosphate-buffered saline (PBS), and viral RNAs were measured by RT-qPCR. (C and D) DEFs were transfected with Flag-duTRIM14 (C) or siduTRIM14-3 (D) for 24 h, followed by incubation with TMUV at 4°C for 1 h. The cells were then washed three times with PBS and incubated at 37°C for 1 h to allow viral entry. Following incubation, the cells were stringently washed with an alkaline high-salt solution, and intracellular viral RNAs were measured using RT-qPCR. (E) TMUV replicon efficiency was assessed using a NanoLuc reporter replicon in BHK-21 cells transfected with Flag-duTRIM14 or treated with the flavivirus RdRp inhibitor HeE1-2Tyr (25 μM). (F and G) Immunoblot analysis was conducted on protein extracts from DEFs transfected with plasmids encoding HA-duTRIM14 and either TMUV structural proteins (F) or nonstructural proteins (G). (H) Immunoblot analysis of protein extracts from DEFs transfected with plasmids encoding Flag-NS1, Flag-NS3, or Flag-NS5, along with increasing amounts of HA-duTRIM14. (I) RT-qPCR analysis of NS1 mRNA levels in DEFs transfected with plasmids encoding Flag-NS1 and increasing amounts of HA-duTRIM14. Results are presented as relative fold changes, normalized to GAPDH controls. (J) Immunoblot analysis of protein extracts from DEFs transfected with plasmids encoding Flag-NS1 and either HA-duTRIM14 or an empty vector for 24 h, followed by treatment with cycloheximide (CHX, 100 μg/ml) for indicated times. (K) Quantification of NS1 protein levels shown in panel (J), with levels normalized to β-actin. Results from RT-qPCR and replicon assays are presented as means ± SD from three independent experiments. Statistical significance was determined by student’s t test (A-D), one-way ANOVA followed by Tukey’s multiple comparisons test (E), or one-way ANOVA followed by Dunett’s multiple comparisons test (I). **, *P* < 0.01; ***, *P* < 0.001; ns, no significant difference.

To elucidate the mechanism underlying duTRIM14’s antiviral activity, we examined its effects on different stages of the TMUV life cycle. Our initial assays confirmed that duTRIM14 does not influence the early stages of TMUV infection, including viral binding and entry (Fig 2A-2D). Using a sub-genomic replicon system that encodes TMUV nonstructural proteins linked to a Nanoluc luciferase (NLuc) reporter, we found that duTRIM14 overexpression significantly reduced TMUV RNA genome replication (Fig 2E). To determine whether this reduction was associated with decreased levels of specific viral proteins essential for replication, we transfected DEFs with plasmids encoding TMUV’s structural proteins (C, prM, or E) or its seven nonstructural proteins (NS1, NS2A, NS2B, NS3, NS4A, NS4B, or NS5), along with HA-tagged duTRIM14. Western blot analysis revealed a specific reduction in TMUV NS1 protein levels due to duTRIM14, while other viral proteins remained unaffected (Fig 2F and 2G). The inhibition of NS1 expression was dose-dependent when co-expressed with duTRIM14, whereas levels of NS3 and NS5 did not change (Fig 2H). RT-qPCR confirmed that duTRIM14 did not alter the mRNA levels of NS1 (Fig 2I). Furthermore, cycloheximide chase (CHX) experiments demonstrated that duTRIM14 promotes the degradation of NS1 protein (Fig 2J and 2K), indicating that duTRIM14 primarily suppresses TMUV replication by reducing the abundance of NS1.

### DuTRIM14 interacts with TMUV NS1 protein

**Fig 3:**
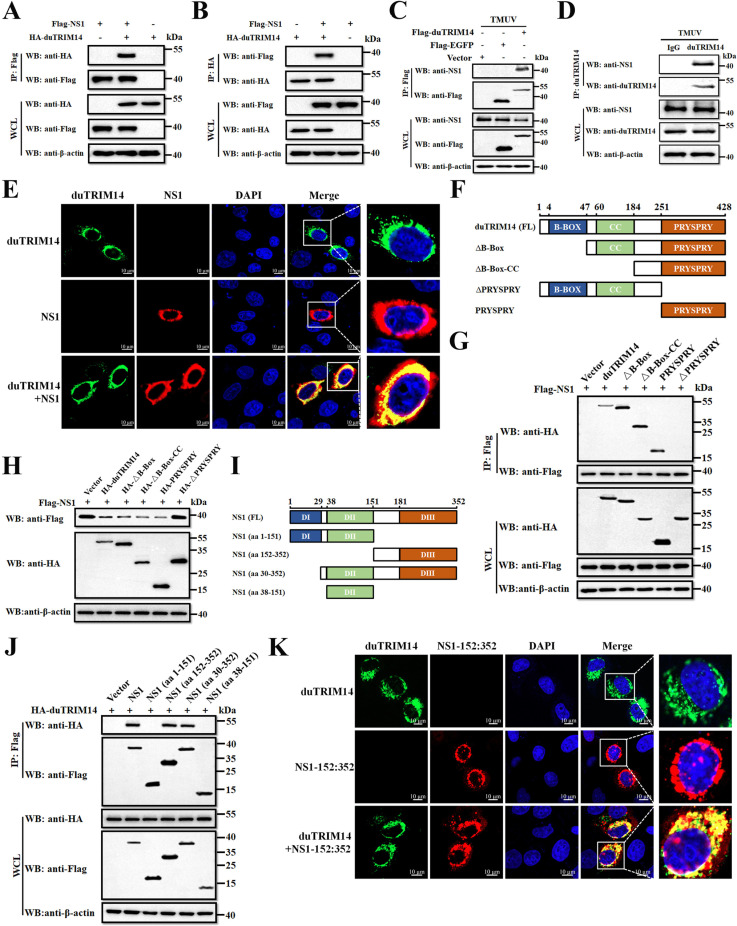
DuTRIM14 interacts with TMUV NS1 protein. (A and B) Lysates from HEK-293T cells co-transfected with Flag-NS1 and HA-duTRIM14 were subjected to Co-IP using anti-Flag (A) and anti-HA (B) antibodies, followed by western blot analysis. (C) Lysates from DEFs transfected with plasmids encoding either Flag-EGFP or Flag-duTRIM14, and subsequently infected with TMUV, were collected for Co-IP using anti-Flag antibody, followed by western blot analysis. (D) Lysates from DEFs infected with TMUV were collected and subjected to co-immunoprecipitation with an anti-duTRIM14 antibody, followed by western blot analysis. (E) HeLa cells were co-transfected with HA-NS1 and Flag-duTRIM14. After 28 h, the cells were fixed and subjected to immunofluorescence staining to detect NS1 (red) using an anti-HA antibody and duTRIM14 (green) using an anti-Flag antibody. Nuclei were stained with DAPI. (F) Schematic diagram of duTRIM14 and its truncate mutants. (G) Co-IP analysis was performed to examine the interactions between Flag-NS1 and HA-tagged duTRIM14 or its truncation mutants in HEK-293T cells. (H) Immunoblot analysis of DEF extracts transfected with plasmids encoding HA-duTRIM14 or its mutants, along with Flag-NS1. (I) Schematic diagram of TMUV NS1 and its truncate mutants. (J) Co-IP analysis was conducted to assess the interactions between Flag-duTRIM14 and HA-tagged NS1, as well as its truncation mutants, in HEK-293T cells. (K) HeLa cells were co-transfected with HA-NS1 (aa 152-352) and Flag-duTRIM14. After 28 h, the cells were fixed and subjected to immunofluorescence staining to detect NS1 (aa 152-352) (red) using an anti-HA antibody and duTRIM14 (green) using an anti-Flag antibody. Nuclei were stained with DAPI.

To understand how duTRIM14 reduces the abundance of TMUV NS1, we first investigated their interaction. HEK293T cells were co-transfected with plasmids encoding HA-duTRIM14 and Flag-NS1, followed by co-immunoprecipitation (co-IP) assays using anti-Flag antibodies. As expected, our results confirmed that duTRIM14 interacted with NS1 (Fig 3A). Reciprocal Co-IP experiments further validated this interaction (Fig 3B), and native NS1 from TMUV-infected DEFs was also precipitated using Flag-duTRIM14 (Fig 3C). More importantly, endogenously expressed duTRIM14 also interacts with native NS1 in TMUV-infected DEFs (Fig 3D). Additionally, confocal microscopy revealed colocalization of duTRIM14 and NS1 in the cells (Fig 3E), demonstrating that duTRIM14 can indeed interact with NS1.

To identify the specific domain responsible for this interaction, we constructed four truncated mutants of duTRIM14: ΔB-BOX (aa 48–428), ΔB-BOX-CC (aa 185–428), ΔPRYSPRY (aa 1–251), and PRYSPRY (aa 252–428) (Fig 3F). Co-IP results indicated that deletion of the C-terminal PRYSPRY domain abolished the interaction with NS1, while the isolated PRYSPRY domain was sufficient for binding (Fig 3G). Notably, the isolated PRYSPRY domain alone was sufficient for NS1 degradation, highlighting its critical role in duTRIM14-mediated NS1 degradation (Fig 3H). To identify the critical region of NS1 required for interaction with duTRIM14, we generated four NS1 mutants: NS1 (aa 1–151), NS1 (aa 152–352), NS1 (aa 30–352), and NS1 (aa 38–151) (Fig 3I). Co-IP assays showed that NS1 mutants containing the aa 152–352 region in the C-terminal retained their ability to interact with duTRIM14, whereas truncated mutants lacking this region lost this ability (Fig 3J). Immunofluorescence staining further confirmed that the NS1 aa 152–352 region co-localized with duTRIM14 (Fig 3K). Molecular docking using Alphafold3 identified key interactions between the PRYSPRY domain of duTRIM14 and NS1, with residues K43, R90, D104, E106, and R166 of duTRIM14 interacting with residues E157, D158, G162, K167, and T174 of NS1 (S3 Fig). These results indicate that the C-terminal region (aa 152–352) of NS1 is crucial for its interaction with duTRIM14.

### DuTRIM14 promotes proteasomal degradation of TMUV NS1 protein

**Fig 4:**
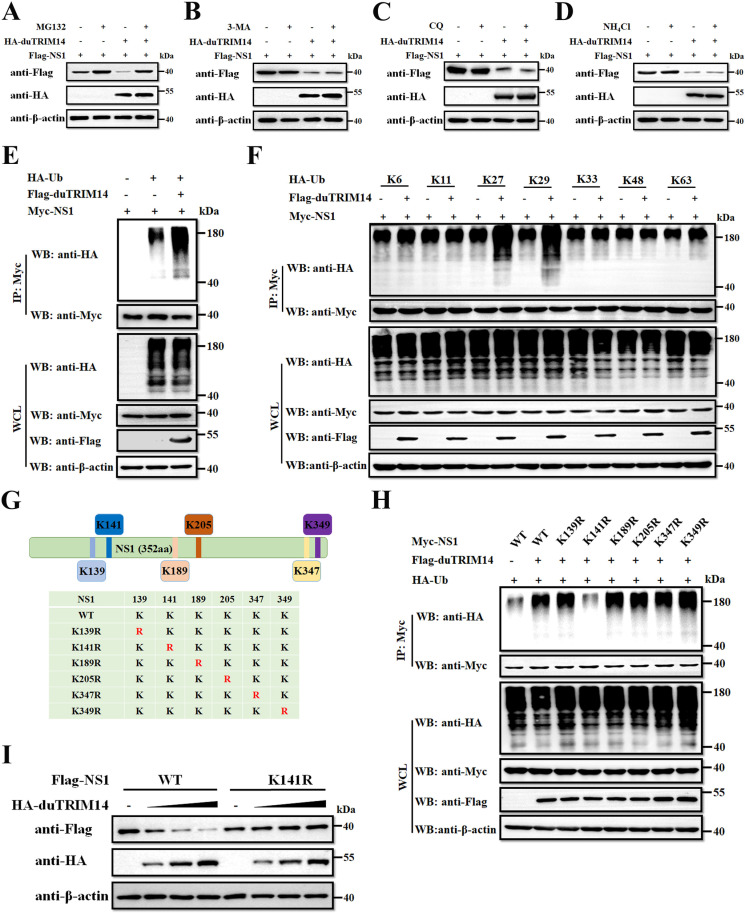
DuTRIM14 promotes proteasomal degradation of TMUV NS1 protein. (A-D) Immunoblot analysis of lysates from DEFs co-transfected with Flag-NS1 and HA-duTRIM14 for 24 h, followed by treatment with various inhibitors: MG-132 (10 µM) (A), 3-MA (10 mM) (B), chloroquine (CQ, 20 µM) (C), or NH_4_Cl (20 mM) (D). (E and F) Lysates from DEFs transfected with Myc-NS1 and HA-tagged ubiquitin (Ub) (E) or its mutants (F), along with either an empty vector or Flag-duTRIM14 in the presence of MG132 (10 µM), were immunoprecipitated using an anti-Myc antibody, followed by western blot analysis. (G) A schematic illustration depicting the potential ubiquitination site on NS1. (H) Co-IP and immunoblot analysis of lysates from DEFs transfected with either Myc-NS1 or its mutants, along with HA-Ub and either an empty vector or Flag-duTRIM14, in the presence of MG132 (10 µM). (I) Immunoblot analysis of lysates from DEFs co-transfected with either Flag-NS1 or its mutant Flag-NS1-K141R, along with increasing amounts of HA-duTRIM14.

To investigate the mechanism by which duTRIM14 facilitates the degradation of TMUV NS1, we aimed to determine whether the ubiquitin-proteasome system (UPS) or the autophagy-lysosomal pathway is predominantly utilized. To this end, DEFs were transfected with plasmids encoding HA-duTRIM14 and Flag-NS1, followed by treatment with proteasome and autophagy inhibitors. Our results showed that NS1 degradation induced by duTRIM14 was completely inhibited by the proteasome inhibitor MG132, while autophagy inhibitors such as 3-MA, CQ, and NH_4_Cl had no effect (Fig 4A-4D). These findings suggest that duTRIM14 mediates NS1 degradation primarily through the ubiquitin-proteasome pathway. Previous studies have shown that protein ubiquitination is a critical step in the ubiquitin-proteasome degradation pathway in eukaryotic cells [32,33]. To elucidate the role of duTRIM14 in NS1 ubiquitination, DEFs were transfected with plasmids encoding Myc-NS1, HA-ubiquitin, and either Flag-duTRIM14 or an empty vector. As shown in Fig 4E, the level of NS1 ubiquitination was significantly enhanced in cells expressing duTRIM14. To further characterize the type of polyubiquitination chains associated with NS1, we co-transfected plasmids expressing the seven ubiquitin mutants (K6, K11, K27, K29, K33, K48, or K63), along with Flag-duTRIM14, and Myc-NS1. This analysis revealed that duTRIM14 increased NS1 ubiquitination specifically in the presence of K27 or K29 ubiquitin (Fig 4F), indicating that duTRIM14 mediates the K27- and K29-linked polyubiquitination of NS1.

Ubiquitination involves the transfer of a ubiquitin molecule to a lysine (K) residue on a target protein [34,35]. To identify the specific residues on NS1 involved in duTRIM14-mediated ubiquitination, we predicted potential ubiquitination sites (K139, K141, K189, K205, K347, and K349) using UBPRED software [30,36]. Each lysine residue was substituted with arginine (R) to assess its role in NS1 ubiquitination (Fig 4G). The K-to-R substitution preserves the positive charge of the residue but eliminates the ε-amino group required for the formation of the ubiquitin linkage, thereby preventing ubiquitination [37,38]. As shown in Fig 4H, only the K141R substitution significantly impaired NS1 ubiquitination mediated by duTRIM14. Furthermore, increasing amounts of duTRIM14 were unable to degrade the K141R NS1 mutant (Fig 4I), indicating that K141 is a critical residue for the ubiquitination and subsequent degradation of NS1 by duTRIM14.

### Removal of the NS1 ubiquitination site at Lys141 enhances TMUV replication

**Fig 5:**
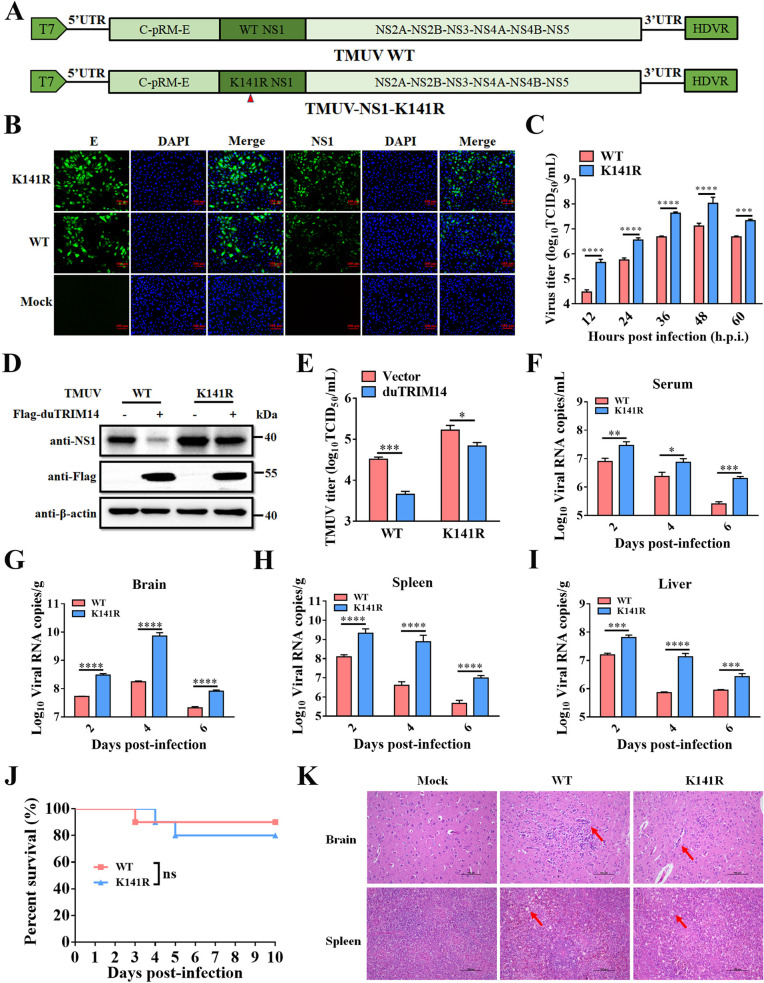
Removal of the NS1 ubiquitination site at Lys141 enhances TMUV replication. (A) Diagram of the TMUV rescue strategy. (B) DEFs were infected with WT or K141R viruses and then subjected to immunofluorescence assay using anti-TMUV E and NS1 antibodies at 24 h post-infection. Scale bars: 100 μm. (C) DEFs were infected with WT or K141R viruses, and the supernatant was collected at different time points for TCID_50_ measurement. Data from TCID_50_ assays are presented as means ± SD from three independent experiments. Statistical significance was determined by two-way ANOVA followed by Sidak’s multiple comparisons test. ***, *P* < 0.001; ****, *P* < 0.0001. (D and E) DEFs were transfected with Flag-duTRIM14 and infected with WT or K141R viruses. At 24 h post-infection, the cells were harvested for western blot analysis (D), and the supernatant was collected for TCID_50_ measurement (E). Data from TCID_50_ assays are presented as means ± SD from three independent experiments. Statistical significance was determined by two-way ANOVA followed by Sidak’s multiple comparisons test. *, *P* < 0.05; ***, *P* < 0.001. (F-I) Viral RNA copies in the serum (F), brain (G), spleen (H), and liver (I) of ducks infected with WT or K141R viruses were quantified by RT-qPCR at 2-, 4-, and 6-days post-infection (dpi). Data represent means ± SD from three ducks. Statistical significance was determined by two-way ANOVA followed by Sidak’s multiple comparisons test. *, *P* < 0.05; **, *P* < 0.01; ***, *P* < 0.001; ****, *P* < 0.0001. (J) Three-day-old SPF ducks were infected with equal doses of WT or K141R viruses, and survival was monitored daily for 10 days. Survival curves were analyzed using a log-rank test (ns, no significant difference). (K) Histopathological examination of brain and spleen tissues from ducks infected with WT and K141R viruses.

To assess the importance of the Lys141 residue in NS1 for duTRIM14-mediated inhibition of TMUV replication, we generated a K141-mutant virus (TMUV-K141R) that lacks this critical ubiquitination site using an infectious cDNA clone of the TMUV strain MC [39] (Fig 5A). Sequencing confirmed the successful mutation of Lys141 to arginine and verified that no unintended mutations were present in the TMUV-K141R genome (S4 Fig). The infectivity of this mutant virus in DEF cells was validated through indirect immunofluorescence assays, which detected viral E and NS1 proteins, confirming the successful rescue of TMUV-K141R (Fig 5B). We then compared the replication kinetics of TMUV-K141R with the wildtype (WT) virus in DEFs, harvesting samples at various post-infection time points. Growth curves indicated that viral titers for TMUV-K141R increased by 4.5-15.1-fold from 12 to 60 h p.i. compared to the WT virus (Fig 5C). Given that duTRIM14 primarily mediates NS1 degradation through ubiquitination at K141, we evaluated its ability to reduce NS1 levels in cells infected with TMUV-K141R versus those infected with WT TMUV. As anticipated, NS1 degradation was significantly reduced in TMUV-K141R-infected DEFs compared to WT-infected cells (Fig 5D). Consequently, the antiviral effects of duTRIM14 were markedly diminished against TMUV-K141R compared to WT TMUV (Fig 5E). These findings indicate that the K141 residue of NS1 is crucial for duTRIM14-mediated suppression of TMUV replication.

To further investigate the impact of Lys141 of NS1 on TMUV replication *in vivo*, three-day-old SPF ducks were inoculated intramuscularly with either the WT or K141R viruses. Three ducks from each group were euthanized at 2, 4, and 6 days post-inoculation (dpi) to assess viral loads in sera and various tissues. As shown in Fig 5F-5I, viral loads in the sera, brain, spleen, and liver from ducks infected with the K141R virus were significantly higher at different time points compared to those infected with the WT virus. To examine the effect of Lys141 on the virulence of TMUV, SPF ducks were infected with both the K141R and WT viruses. Unexpectedly, the mortality rates in the K141R-infected ducks did not significantly differ from those in the WT-infected group (Fig 5J). Additionally, histopathological analysis revealed that the K141R mutant induced comparable lesions in the brain and spleen of SPF ducks, similar to those caused by the WT virus (Fig 5K). These lesions included vascular cuffing in the brain, as well as lymphocyte apoptosis in the spleen (Fig 5K). Collectively, these findings indicate that the removal of the NS1 ubiquitination site at Lys141 enhances TMUV replication *in vivo*, but does not affect viral virulence.

### DuTRIM14 enhances the RLR signaling pathway

**Fig 6:**
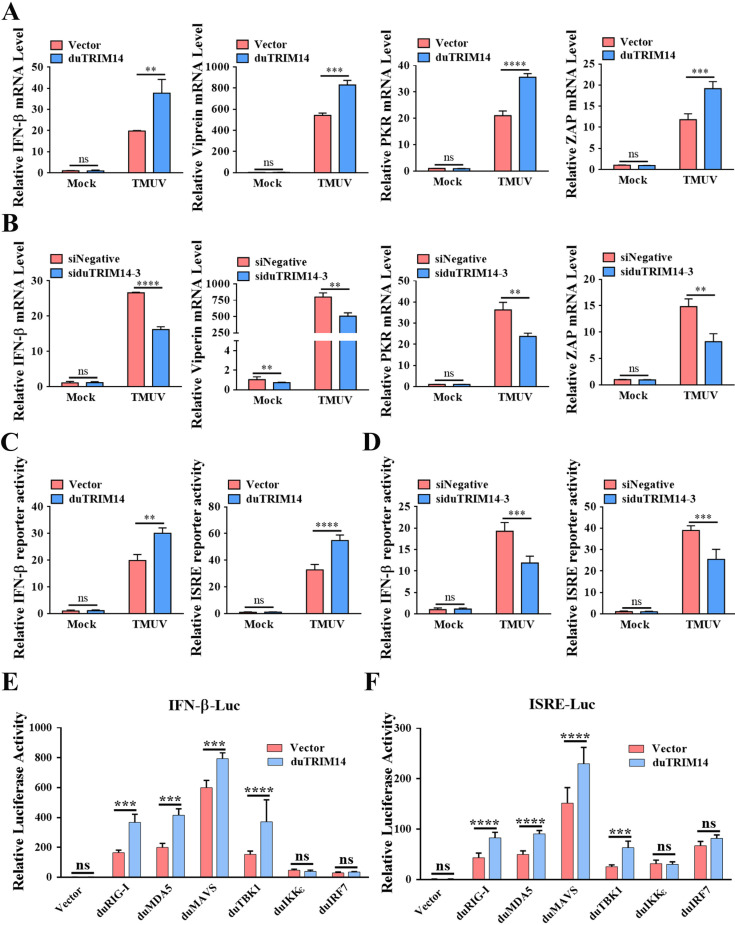
DuTRIM14 enhances the RLR signaling pathway. (A and B) DEFs were transfected Flag-duTRIM14 (A) or siduTRIM14-3 (B), followed by TMUV (MOI = 0.1) infection. At 24 h post-infection, relative expression levels of IFN-β, viperin, PKR, and ZAP were assessed by RT-qPCR. (C and D) Luciferase reporter assays were conducted to analyze IFN-β and ISRE promoter activities in DEFs transfected with Flag-duTRIM14 (C) or siduTRIM14-3 (D), followed by TMUV infection. (E and F) Luciferase reporter assays were conducted to analyze the activities of the IFN-β (E) and ISRE (F) promoters in DEFs co-transfected with plasmids encoding key components of the RLR signaling pathway, along with either an empty vector or Flag-duTRIM14. All RT-qPCR results are expressed as relative fold changes, normalized to GAPDH. Results are presented as means ± SD from three independent experiments. Statistical significance was determined by two-way ANOVA followed by Sidak’s multiple comparisons test. *, *P* < 0.05; **, *P* < 0.01; ***, *P* < 0.001; ****, *P* < 0.0001; ns, no significant difference.

Previous studies have shown that several TRIM proteins can inhibit viral infections not only by directly targeting viral proteins but also by enhancing antiviral innate immunity [40,41]. To explore this role for duTRIM14, we investigated the expression of IFN-β and ISGs using RT-qPCR. Our results revealed that the mRNA expression of IFN-β, viperin, PKR, and ZAP induced by TMUV was significantly higher in DEFs expressing duTRIM14 compared to those with an empty vector (Fig 6A). In contrast, knockdown of duTRIM14 led to a notable reduction in TMUV-induced expression of these genes (Fig 6B). Furthermore, luciferase assays showed that duTRIM14 expression significantly enhanced the activation of IFN-β and ISRE promoters in response to TMUV (Fig 6C). Conversely, the knockdown of duTRIM14 resulted in a marked decrease in the activity of these promoters during TMUV infection (Fig 6D). These results suggest that duTRIM14 expression amplifies TMUV-induced IFN-β and ISGs expression. Given the critical role of RLR-mediated IFN-β production in host defense against RNA virus infection, we further examined which components of the duck RLR signaling pathway are regulated by duTRIM14 through luciferase reporter assays. We observed that duTRIM14 enhanced the activation of duIFN-β or ISRE promoters triggered by duRIG-I, duMDA5, duMAVS, and duTBK1, but not by duIKKε or duIRF7 (Fig 6E and 6F). This pattern suggests that duTRIM14 may specifically enhance IFN-β production by targeting duTBK1 within the RLR signaling cascade.

### DuTRIM14 promotes K63-linked polyubiquitination of TBK1

**Fig 7:**
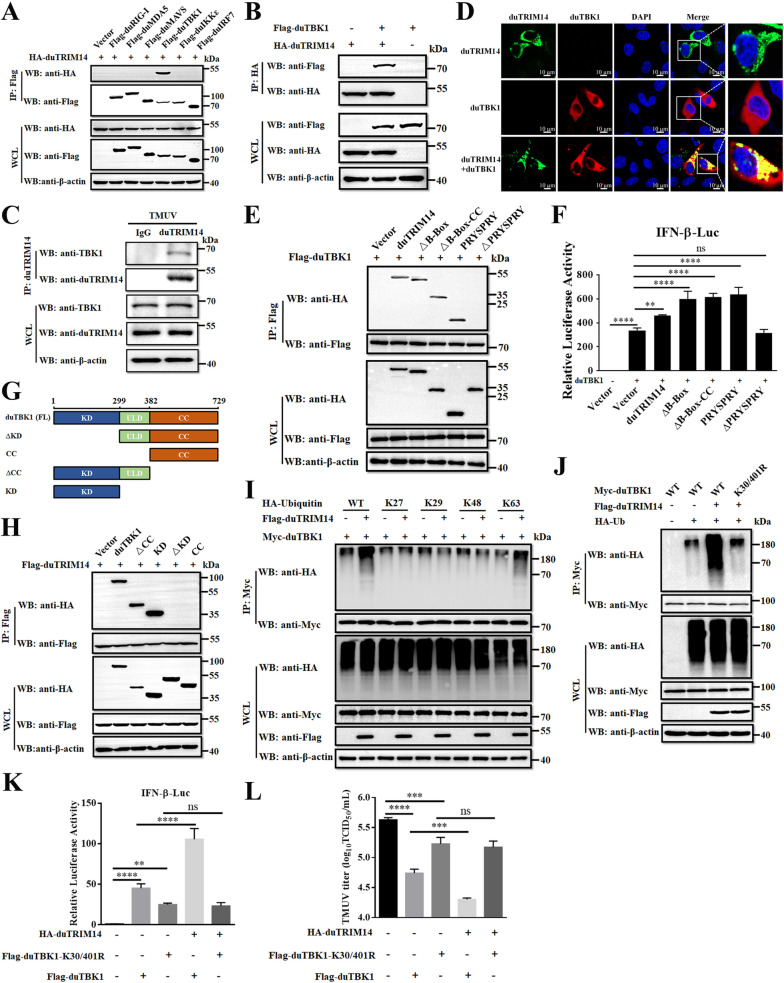
DuTRIM14 promotes K63-linked polyubiquitination of TBK1. (A) Lysates from HEK-293T cells co-transfected with HA-duTRIM14 and Flag-tagged duRIG-I, duMDA5, duMAVS, duTBK1, duIKKε, or duIRF7 were subjected to Co-IP using an anti-Flag antibody, followed by western blot analysis. (B) Co-IP analysis of lysates from HEK-293T cells transfected with HA-duTRIM14 and Flag-duTBK1 using an anti-HA antibody. (C) Lysates from DEFs infected with TMUV were collected and subjected to co-immunoprecipitation with an anti-duTRIM14 antibody, followed by western blot analysis. (D) HeLa cells co-transfected with HA-duTBK1 and Flag-duTRIM14 were fixed and subjected to immunofluorescence assays. DuTBK1 was detected with an anti-HA antibody (red), and duTRIM14 with an anti-Flag antibody (green). Nuclei were stained with DAPI. (E) Co-IP analysis of the interaction between Flag-duTBK1 and HA-duTRIM14 or its truncation mutants in HEK-293T cells. (F) Luciferase reporter assays were conducted to analyze IFN-β promoter activity in DEFs co-transfected with plasmids encoding HA-duTRIM14 or its mutants, along with Flag-duTBK1. (G) Schematic diagram of duTBK1 and its truncate mutants. (H) Co-IP analysis of the interaction between Flag-duTRIM14 and HA-tagged duTBK1 or its truncation mutants in HEK-293T cells. (I) Lysates from DEFs co-transfected with Myc-duTBK1 and HA-Ub or its mutants, along with either an empty vector or Flag-duTRIM14 in the presence of MG132 (10 μM), were immunoprecipitated with an anti-Myc antibody, followed by western blot analysis. (J) Co-IP analysis of lysates from DEFs transfected with Myc-duTBK1 or its mutant, together with HA-Ub and either Flag-duTRIM14 or an empty vector, in the presence of MG132 (10 μM). (K) Luciferase reporter assays were conducted to analyze IFN-β promoter activity in DEFs co-transfected with plasmids encoding Flag-duTBK1 or its mutants, along with HA-duTRIM14. (L) DEFs were co-transfected with Flag-duTBK1 or Flag-duTBK1-K30/401R, along with HA-duTRIM14, followed by infection with K141R viruses. At 24 h post-infection, the cell supernatant was collected for TCID_50_ measurement. Data from luciferase reporter and TCID_50_ assays are presented as means ± SD from three independent experiments. Statistical significance was determined by one-way ANOVA followed by Dunett’s multiple comparisons test (F), or one-way ANOVA followed by Tukey’s multiple comparisons test (K and L). **, *P* < 0.01; ***, *P* < 0.001; ****, *P* < 0.0001; ns, no significant difference.

To determine whether duTRIM14 targets duTBK1 to enhance RLR signaling, co-IP experiments were conducted. These experiments revealed that duTRIM14 specifically associates with duTBK1, while showing no interaction with duRIG-I, duMDA5, duMAVS, duIKKε, or duIRF7 (Fig 7A). This specificity was further corroborated by reverse co-IP assays (Fig 7B). Importantly, an interaction between endogenous duTRIM14 and duTBK1 was also observed in TMUV-infected DEFs (Fig 7C). Additionally, confocal microscopy confirmed the colocalization of duTRIM14 and duTBK1 in the cytoplasm (Fig 7D). To identify the critical domain of duTRIM14 responsible for this interaction, co-IP assays showed that removal of the C-terminal PRYSPRY domain abolished the interaction with duTBK1, whereas the isolated PRYSPRY domain was sufficient for binding (Fig 7E). Consistent with these results, the isolated PRYSPRY domain retained its ability to enhance duIFN-β activation mediated by duTBK1 (Fig 7F). Further Co-IP assays using truncated mutants of duTBK1 identified the kinase domain (KD) of duTBK1 as essential for interaction with duTRIM14 (Fig 7G and 7H).

K63-linked ubiquitination of TBK1 is crucial for its activation and the subsequent expression of type I IFN [42,43]. Therefore, we examined the effect of duTRIM14 on duTBK1 ubiquitination. Immunoprecipitation assays revealed that overexpression of duTRIM14 significantly increased K63-linked ubiquitination of duTBK1, with minimal impact on K27, K29, and K48-linked ubiquitination (Fig 7I). Given that residues K30 and K401 in human TBK1, which are conserved in ducks, are critical for its ubiquitination and catalytic activation (S5 Fig), we generated a Myc-duTBK1 mutant (K30R/K401R) and co-transfected it with Flag-duTRIM14 and HA-Ubiquitin into DEFs. Western blot analysis indicated that duTRIM14 did not enhance ubiquitination in the K30R/K401R mutant (Fig 7J), suggesting that duTRIM14 specifically promotes K63-linked ubiquitination of duTBK1 at residues K30 and K401. Consistently, overexpression of duTRIM14 significantly enhanced IFN-β expression mediated by wild-type duTBK1, but had little effect on IFN-β expression induced by the duTBK1 K30R/K401R mutant (Fig 7K). To further evaluate the role of TRIM14 in duTBK1-mediated antiviral activity, we utilized the mutant virus TMUV-K141R, which eliminates the effect of duTRIM14 on NS1 degradation. The results showed that both wild-type duTBK1 and its K30R/K401R mutant exhibited significant antiviral effects against TMUV-K141R (Fig 7L). However, duTRIM14 expression enhanced the antiviral effect of wild-type duTBK1 against TMUV-K141R, but had no impact on the antiviral activity of the duTBK1-K30R/K401R mutant (Fig 7L). Taken together, these findings demonstrate that TRIM14 not only promotes the proteasomal degradation of NS1 but also enhances duTBK1-mediated antiviral activity by facilitating K63-linked polyubiquitination of duTBK1, indicating an additive effect of these two pathways.

## Discussion

As an emerging avian orthoflavivirus, TMUV poses significant challenges to the poultry industry due to its widespread disease transmission and the resultant economic losses. TMUV infection involves complex interactions between the host and the virus, underscoring the necessity of understanding these dynamics to elucidate the regulatory mechanisms of viral replication and pathogenicity. In this study, we identified duTRIM14 as a novel host restriction factor that combats TMUV infection. Our findings demonstrate that duTRIM14 inhibits TMUV replication by interacting with the viral NS1 protein, leading to K27/K29-linked polyubiquitination and subsequent proteasomal degradation of NS1. Furthermore, we pinpointed Lys141 on NS1 as a critical ubiquitination site; its substitution with arginine disrupted NS1 ubiquitination and degradation, significantly enhancing TMUV replication both *in vitro* and *in vivo*. Additionally, duTRIM14 serves as a positive regulator of RIG-I-mediated antiviral innate immunity by promoting K63-linked ubiquitination of duTBK1, which synergistically inhibits TMUV replication.

TRIM14, initially recognized as KIAA0129, was first detected as being overexpressed in HIV-infected human and simian lymphomas through subtractive hybridization techniques [44–46]. Subsequent research has highlighted TRIM14’s involvement in various biological processes, including cell proliferation, apoptosis, tumor suppression, cancer progression, innate immunity, and viral infection [47–49]. Notably, TRIM14 has exhibited antiviral activity against a range of viruses, including hepatitis C virus (HCV), hepatitis B virus (HBV), Influenza A virus (IAV), and Ebola virus (EBOV) [50–53]. However, despite being the orthologue of human TRIM14, the role of duTRIM14 in modulating TMUV replication remained unexplored. Our study demonstrates that overexpression of duTRIM14 inhibits TMUV replication, while its knockdown results in increased viral titers. Importantly, we found that duTRIM14 significantly reduces TMUV replication without affecting virus adsorption and entry into cells. This observation aligns with recent findings by Li et al., who reported that PARP12 inhibits Zika virus (ZIKV) replication by promoting degradation of ZIKV NS1 and NS3 proteins without hindering virus entry [54]. Similarly, our results indicate that TMUV NS1 interacts with duTRIM14, leading to NS1 degradation in DEFs while not affecting the stability of other proteins. This reduction in TMUV replication may be attributed to the diminished abundance of viral NS1 protein. Moreover, the duTRIM14-mediated degradation of NS1 was nearly completely reversed by MG132 treatment, suggesting that duTRIM14 targets NS1 for degradation via the ubiquitin-proteasome pathway. To our knowledge, this study provides the first evidence of a host protein specifically targeting a TMUV-encoded protein for degradation, thereby revealing a novel mechanism of the host’s antiviral defense.

Flavivirus NS1 is primarily localized within the lumen of the ER, where it forms membrane-associated dimers and contributes to ER membrane remodeling [55,56]. However, recent studies suggest that NS1 may have a broader subcellular distribution beyond the ER. For example, Michita et al. reported that Zika virus NS1 colocalizes with mitochondria in HTR-8 trophoblast cells and interacts with various mitochondrial proteins, as identified by affinity purification-mass spectrometry [57]. These findings imply that flavivirus NS1 may also associate with mitochondria, potentially through organelle contact sites. Consistent with potential localization to specific subcellular compartments, TMUV NS1 in our study exhibited a punctate distribution and colocalized with duTRIM14 in membranous structures. Given that human TRIM14 predominantly localizes to the outer mitochondrial membrane, it is plausible that the interaction between duTRIM14 and TMUV NS1 occurs at mitochondria or within mitochondria-associated membranes (MAMs)-specialized subdomains that mediate dynamic physical and functional interactions between the ER and mitochondria.

TRIM proteins are integral to the regulation of viral replication, primarily through their involvement in the innate immune response. For instance, TRIM5α restricts HIV-1 and other retroviruses by catalyzing the synthesis of unanchored K63 polyubiquitin chains [58,59]. This activity significantly activates various signaling pathways, including TAK1, AP-1, and NF-κB, thereby enhancing the host’s antiviral immune response [58,60,61]. Similarly, TRIM35 enhances RIG-I-mediated antiviral signaling by facilitating the K63-linked polyubiquitination of TRAF3, which promotes the formation of a signaling complex with MAVS, leading to the production of type I interferons and the inhibition of Influenza A virus (IAV) replication [29]. Recently, Hoffpauir et al. found that knockout of mouse TRIM14 results in hyper-induction of type I interferon during *Mycobacterium tuberculosis* infection [49], suggesting an inhibitory role for mouse TRIM14 in innate immunity. In contrast, human TRIM14 acts as an adaptor, facilitating the assembly of the MAVS signaling complex and enhancing antiviral responses [62]. Another report also reveals that human TRIM14 promotes innate immune responses by inhibiting cGAS degradation [63]. In our current study, we demonstrated that duck TRIM14 directly interacts with duTBK1 and catalyzes its K63-linked polyubiquitination, leading to enhanced IFN-β expression and suppression of TMUV replication in DEFs. These results suggests that TRIM14 from different species employs distinct strategies to modulate antiviral innate immune responses. Notably, the amino acid sequence identity between duck and mammalian TRIM14 is approximately 50%, which may contribute to the observed differences in how TRIM14 regulates innate immunity across species.

TRIM proteins regulate viral replication through their E3 ligase activity, which facilitates the ubiquitination of both viral and host proteins. Unlike typical TRIM proteins, duTRIM14 lacks an N-terminal RING domain, suggesting it might not independently catalyze ubiquitination reactions as many conventional E3 ligases do. However, our findings reveal that duTRIM14 facilitates K27/29-linked polyubiquitination of the viral NS1 protein, leading to its proteasomal degradation, as well as K63-linked polyubiquitination of duTBK1, thereby enhancing type I IFN production during TMUV infection. These observations confirm duTRIM14’s critical role in the ubiquitination of viral NS1 and duTBK1. Despite its lack of a RING domain, we hypothesize that duTRIM14 may still play significant roles in these ubiquitination processes by acting as a scaffold to recruit other E3 ligases. This hypothesis is supported by studies on human TRIM14, which interacts with the E3 ubiquitin ligase RNF125; this interaction promotes K48-linked polyubiquitination of human TRIM14 itself, facilitating its degradation via the ubiquitin-proteasome pathway [64]. Notably, our attempts to clone duck RNF125 from a predicted gene sequence in GenBank (XM_038173674.1) were unsuccessful, likely due to premature translation termination and less than 50% amino acid homology with its human counterpart. These challenges indicate potential inaccuracies in the predicted sequence. Consequently, further research is needed to identify the specific E3 ligases that duTRIM14 recruits for the ubiquitination of viral NS1 and duTBK1.

Orthoflavivirus NS1 plays a multifaceted role in viral replication, contributing to the formation of the viral replication complex within the ER. It interacts with other nonstructural proteins, such as NS4A and NS4B, to facilitate RNA synthesis [9,65,66]. Additionally, NS1’s interaction with lipid membranes is crucial for its replication and immune modulation functions [67,68]. Our research demonstrates that duTRIM14 enhances the degradation of NS1 by facilitating its ubiquitination at the K141 site, effectively inhibiting TMUV replication. Utilizing a reverse genetics approach, we mutated the K141 site of NS1 and observed that this modification significantly increased TMUV replication both *in vivo* and *in vitro*. However, our animal experiments indicated that the removal of this ubiquitination site had minimal impact on viral pathogenicity. These findings indicate that while the NS1 K141 mutation enhances TMUV replication and viral titers, it does not necessarily correlate with increased pathogenicity. This aligns with previous studies, such as Wang et al.’s report that mutation of the Lys854 ubiquitination site in IBDV VP3 significantly enhanced viral replication both *in vitro* and *in vivo* without altering virulence [30]. Similarly, certain SARS-CoV-2 variants exhibit increased transmissibility and replication capacity but do not correlate with heightened disease severity [69]. These observations suggest that viral pathogenicity is not solely determined by replication levels but is also influenced by other factors, such as the host immune response, the route of infection, and environmental conditions. Given the current reliance on inactivated and live-attenuated vaccines for TMUV prevention and control, engineering a recombinant TMUV with a mutation at the NS1 K141 site presents an optimal strategy to enhance virus titers. This approach could provide critical insights for developing high-titer, cost-effective vaccines to combat TMUV infection.

**Fig 8:**
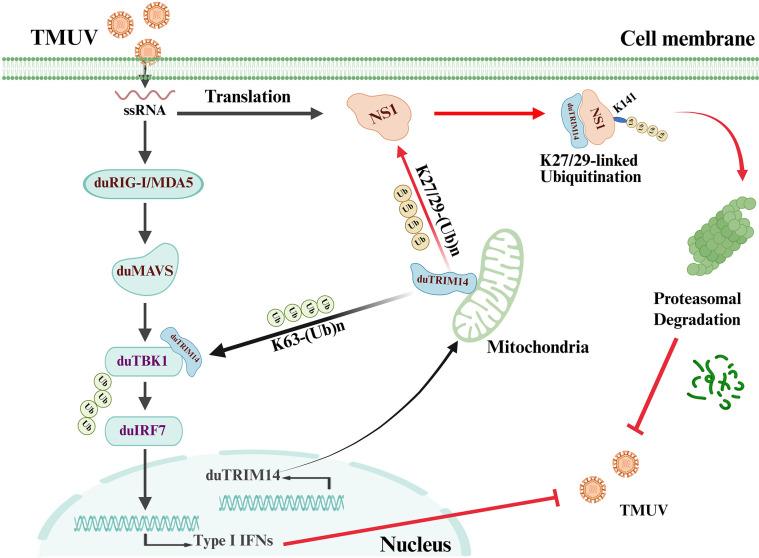
Proposed model illustrating the antiviral mechanism of duTRIM14. During TMUV infection, duTRIM14 specifically targets the TMUV NS1 protein, promoting its K27/29-linked ubiquitination at Lys141, leading to proteasomal degradation of NS1 and inhibition of TMUV replication. Additionally, duTRIM14 catalyzes K63-linked polyubiquitination of duTBK1, which activates the phosphorylation and nuclear translocation of IRF7. This process triggers the IFN-β signaling pathway, further suppressing viral replication. Created in BioRender.

In summary, our study establishes duTRIM14 as a key host restriction factor against TMUV. Mechanistically, duTRIM14 specifically targets the TMUV NS1 protein, facilitating its K27/29-linked ubiquitination at Lys141, leading to proteasomal degradation of NS1. Mutations at this critical lysine residue impair the ubiquitination process, resulting in enhanced TMUV replication both *in vitro* and *in vivo*. Furthermore, duTRIM14 also catalyzes K63-linked polyubiquitination of duTBK1, activating the host’s innate antiviral defense mechanisms (Fig 8). Leveraging these intrinsic defense mechanisms could provide more effective approaches for controlling TMUV infections. More importantly, our research lays the foundation for future studies investigating the broader role of TRIM proteins in antiviral immunity in different species. The unique functionality of duTRIM14 compared to its mammalian counterparts underscores the importance of species-specific research in understanding and combating viral infections. As we continue to unravel the complex interactions between host and virus, this work represents an important step towards the development of novel host-targeted antiviral therapies and more effective vaccines against TMUV and potentially other orthoflaviviruses.

## Materials and methods

### Ethics statement

This study was conducted in strict adherence to the guidelines outlined in the “Guide for the Care and Use of Laboratory Animals” issued by the Ministry of Science and Technology of the People’s Republic of China. All animal experiments received approval from the Scientific Ethics Committee of Huazhong Agricultural University, Hubei, China (Approval No. HZAUDU-2022–0003).

### Cells and viruses

Duck embryo fibroblasts (DEFs) were cultured in Minimum Essential Medium (MEM, Gibco, C12571500BT) supplemented with 10% fetal bovine serum (Gibco, 16000044). Human embryonic kidney cells (HEK293T), human cervical cancer cell line (HeLa), and Baby hamster kidney cells (BHK-21) were maintained in Dulbecco’s Modified Eagle’s Medium (DMEM, Gibco, 12800082) supplemented with 10% FBS. The TMUV strain MC (GenBank accession number: KX452096) was preserved in our laboratory. A recombinant virus was generated based on the genetic background of the TMUV MC strain using a four-plasmid-based reverse genetics system, as previously described [39]. TMUV titers were determined in DEFs using a median tissue culture infective dose (TCID_50_) assay, calculated according to the Reed-Muench method.

### Antibodies and reagents

Mouse monoclonal antibodies against Flag (M185-3L), HA (M180-3), and Myc (M192-3) were obtained from MBL International Corporation. Rabbit polyclonal antibodies against Flag (PM020) and HA (PM032) were also sourced from MBL International Corporation. Rabbit anti-β-actin monoclonal antibody (AC038) was obtained from ABclonal Technology. The monoclonal antibody against duTRIM14 was generated by immunizing BALB/c mice with purified recombinant duTRIM14 protein expressed in *Escherichia coli*. The anti-TMUV envelope (E) monoclonal antibody (clone 3F12) was prepared previously in our laboratory, while the anti-TMUV NS1 monoclonal antibody was kindly provided by Professor Shun Chen from Sichuan Agricultural University, Chengdu, China. Horseradish peroxidase (HRP)-conjugated goat anti-rabbit or anti-mouse secondary antibodies were purchased from ABclonal Biotechnology (AS003 and AS014). HRP-Goat Anti-Mouse IgG LCS (A25012) were purchased from Abbkine. Alexa Fluor 488-labeled goat anti-mouse IgG (A-11001) and Alexa Fluor 594-labeled goat anti-rabbit IgG (A-11012) were sourced from Invitrogen. Protein A/G Plus-Agarose (sc-2003) was acquired from Santa Cruz Biotechnology. The orthoflavivirus RNA-dependent RNA polymerases (RdRp) inhibitor HeE1-2Tyr (HY-100749) was obtained from MedChemExpress. MG132 (M7449) and chloroquine phosphate (PHR1258) were obtained from Sigma-Aldrich. NH_4_Cl (ST2030) and 3-methyladenine (3-MA, S2767) were purchased from Beyotime Biotechnology and Selleck Chemicals, respectively.

### Plasmids, small interfering RNAs (siRNAs) and transfections

Eukaryotic expression plasmids containing genes for 20 distinct duck TRIM proteins were constructed by cloning individual TRIM cDNA from DEFs into the pCAGGS-Flag vector. The TMUV protein-encoding genes were cloned into either the pCAGGS-HA or Flag vector as previously described [70]. Flag-tagged constructs for duRIG-I, duMDA5, duMAVS, duTBK1, duIKKε, and duIRF7 were prepared in our laboratory following established protocols [71]. TRIM14 mutants (aa 48–428, aa 185–428, aa 1–251 and aa 252–428) and truncated TMUV NS1 mutants (aa 1–151, aa 152–352, aa 30–352, and aa 38–151) were subcloned into the pCAGGS expression vector with an N-terminal HA tag. Full-length and truncated versions of duTBK1 and TMUV NS1 were also cloned into pcDNA3.1 with a Myc tag. Site-directed mutagenesis was used to generate point mutants of Myc-NS1 (K139R, K141R, K189R, K205R, K347R, and K349R) and Myc-TBK1 (K30/401R), as previously described [70]. The Nanoluc luciferase reporter replicon of TMUV was kindly provided by Professor Shun Chen (Sichuan Agricultural University, Chengdu, China). The IFN-β-Luc reporter plasmid has been described in our previous report [71]. The ISRE-Luc reporter construct was obtained from Promega (Madison, WI, USA). All primers used are listed in S1 Table. SiRNAs targeting specific genes were designed and synthesized by GenePharma (China) (S2 Table). Transfections of plasmids or siRNA into DEF, HEK293T, or HeLa cells were performed using Lipofectamine 2000 (Invitrogen).

### Luciferase reporter assays

DEFs cultured in 48-well plates were co-transfected with 50 ng/well of luciferase reporter plasmid and 50 ng/well of Renilla luciferase-expressing plasmid (pRL-TK, Promega, USA), along with the indicated expression plasmids, using Lipofectamine 2000. At 24 h post transfection, cells were either infected with TMUV. Cells were lysed in passive lysis buffer (Promega, E1941), and luciferase activity was measured using the Dual-Luciferase Reporter Assay System (Promega, E1910) according to manufacturer’s instructions. Results were expressed as relative firefly luciferase activities normalized to Renilla luciferase activities. All experiments were performed in triplicate.

### RT-qPCR

Total RNA was extracted from treated DEFs using TRIzol reagent (Invitrogen, USA) and reverse-transcribed with the Transcriptor First Strand cDNA Synthesis Kit (Roche, Switzerland). RT-qPCR was performed using the FastStart Universal SYBR Green Master Mix (Roche, Switzerland) on an Applied Biosystems 7500 Fast Real-Time PCR System. Transcript levels were normalized to duck glyceraldehyde-3-phosphate dehydrogenase (GAPDH) and analyzed using the ΔΔCt method.

### Fluorescence microscopy

HeLa cells were seeded onto coverslips placed in 12-well plates and transfected with specific expression plasmids upon reaching approximately 50% confluence. Following transfection, cells were fixed with 4% paraformaldehyde for 20 minutes, then permeabilized with 0.1% Triton X-100 for 15 minutes at room temperature. Cells were subsequently blocked with 5% bovine serum albumin (BSA) for 1 h, followed by incubation with primary antibodies for 1 h. After three washes with PBS-Tween (PBST), cells were stained with Alexa Fluor-conjugated secondary antibodies for 1 h, and nuclei were counterstained with 4′,6-diamidino-2-phenylindole (DAPI) for 10 minutes at room temperature. Imaging was performed using a Zeiss LSM 880 confocal microscope.

### Western blot and Co-IP analysis

For western blot analysis, DEFs were lysed in lysis buffer (65 mM Tris-HCl, 4% sodium dodecyl sulfate, 3% DL-dithiothreitol, 40% glycerol, and protease inhibitors including 1 mM phenylmethanesulfonyl fluoride [PMSF]). Equal amounts of cell lysates were denatured, separated by 12% SDS-PAGE, and transferred onto polyvinylidene fluoride (PVDF) membranes (Millipore, USA). Membranes were blocked with 5% nonfat milk, incubated with primary and HRP-conjugated secondary antibodies. Detection was performed using an enhanced chemiluminescence (ECL) system (Bio-Rad, USA).

For the Co-IP assay, HEK293T cells or DEFs were co-transfected with the indicated plasmids, washed with ice-cold PBS, and lysed in 500 μL ice-cold RIPA buffer (P0013B, Beyotime, China) supplemented with a protease inhibitor cocktail (P8340, Sigma) for 30 min. After centrifugation at 13,000 × g for 10 minutes, the supernatant was incubated overnight at 4°C with specific antibodies with agitation. This was followed by incubation with protein A/G plus agarose (sc-2003, Santa Cruz Biotechnology) for 4 hours at 4°C. The beads were collected by centrifugation at 2,500 × g for 5 minutes at 4°C, washed four times with cold PBST, and the bound proteins were eluted with SDS loading buffer by boiling for 10 minutes. The proteins were then analyzed using standard immunoblotting procedures.

### Ubiquitination assay

To evaluate the effect of duTRIM14 on NS1 ubiquitination, HEK293T cells were co-transfected with Flag-NS1, HA-Ub, and either Myc-duTRIM14 or an empty vector. At 24 h post-transfection, cells were treated with MG132 for 6 h before being harvested with RIPA buffer (P0013B, Beyotime, China) containing protease inhibitor cocktail (P8340, Sigma). Cell lysates were immunoprecipitated using anti-Flag mAb and analyzed by immunoblotting with anti-HA, anti-Myc, and anti-Flag antibodies. A similar protocol was employed to investigate the influence of duTRIM14 on the ubiquitination of duTBK1.

### Reverse genetics

The NS1 site-directed mutant virus, TMUV-NS1-K141R, was generated using a previously described reverse genetic system [39]. Briefly, site-directed mutagenesis was performed using the Q5 site-directed mutagenesis kit (NEB, Ipswich, MA, USA) and confirmed by Sanger sequencing. Following digestion, purification, and ligation, full-length cDNA of TMUV-NS1-K141R was assembled. In vitro RNA synthesis was conducted using the mMESSAGE mMACHINE T7 Transcription Kit (Ambion) and subsequently electroporated into DEFs at 450 V and 50 µF using the Gene Pulser X cell electroporation system (Bio-Rad). The rescued TMUV-NS1-K141R virus was harvested and purified via plaque assay.

### Animal experiments

For pathogenicity comparison, groups of 3-day-old SPF ducks were injected intramuscularly with 10^7^ TCID_50_ of either wild-type TMUV or its K141R mutant; control ducks received an equivalent volume of MEM medium. Ducks showing signs of apparent mental depression were humanely euthanized. Survival rates and weight changes were monitored over 10 days. For replication comparisons, ducks were inoculated as described and euthanized at 2, 4, and 6 days post-inoculation (dpi) for sample collection from serum, brain, spleen, and liver to determine virus titers. At the conclusion of the study, the remaining ducks were humanely euthanized by intravenous injection of sodium pentobarbital at a dosage of 100 mg/kg body weight.

### Statistical analysis

Data were analyzed using GraphPad Prism software. The significance of differences was analyzed with Student’s t-test, One-way ANOVA test, or Two-way ANOVA test. Survival percent was analyzed by log-rank (Mantel-Cox) test. Data are presented as mean ± standard deviation (SD). *, *P* < 0.05; **, *P* < 0.01; ***, *P* < 0.001; ****, *P* < 0.0001.

## Supporting information

S1 Fig TMUV infection induces the expression of duTRIM14 in DEFs. (A and B) DEFs were inoculated with TMUV at an MOI of 0.1 for the indicated time points. RT-qPCR and Western blotting were used to measure duTRIM14 protein and mRNA levels. Results from RT-qPCR are presented as means ± SD from three independent experiments. Statistical significance was determined by two-way ANOVA followed by Sidak’s multiple comparisons test (****P < 0.0001).
(DOCX)

S2 Fig Gene silencing efficiency of siRNA targeting duTRIM14 in DEFs. DEFs were transfected with either control siRNA (siNegative) or siRNA targeting duTRIM14 (siduTRIM14). (A) At 30 h post-transfection, intracellular mRNA was extracted and the abundance of duTRIM14 mRNA was quantified by RT-qPCR. Data are representative of three independent experiments and presented as mean ± SD. Statistical significance was determined by one-way ANOVA followed by Dunett’s multiple comparisons test (***P < 0.001, ****P < 0.0001). (B) Immunoblot analysis of lysates from DEFs transfected with siNegative or siduTRIM14 with an anti-duTRIM14 antibody.
(DOCX)

S3 Fig Molecular docking prediction results for duTRIM14-PRYSPRY and TMUV NS1 proteins. (DOCX)

S4 Fig Confirmation of TMUV NS1-K141R mutant virus by Sanger sequencing. (DOCX)

S5 Fig Alignment of TBK1 protein sequences between human and duck. The protein sequences of TBK1 between human and duck were compared and analyzed with ESPript 3.0 online software (https://espript.ibcp.fr/ESPript/cgi-bin/ESPript.cgi).
(DOCX)

S1 Table Primers for RT-qPCR and plasmid construction in this study. (DOCX)

S2 Table The sequences of siRNAs used in the study. (DOCX)

S1 Data The raw data supporting each of the manuscript Figures are contained in this Excel file. (XLSX)
