# Supplementary material for: TRIM14 restricts tembusu virus infection through degrading viral NS1 protein and activating type I interferon signaling
Source: PLoS Pathog. 2025 May 28;21(5):e1013200. doi: 10.1371/journal.ppat.1013200 (PMC12118852; doi:10.1371/journal.ppat.1013200)
Supplement: S1 Fig — (A and B) DEFs were inoculated with TMUV at an MOI of 0.1 for the indicated time points. RT-qPCR and Western blotting were used to measure duTRIM14 protein and mRNA levels. Results from RT-qPCR are presented as means ± SD from three independent experiments. Statistical significance was determined by two-way ANOVA followed by Sidak’s multiple comparisons test (****P < 0.0001). (DOCX) [file ppat.1013200.s001.docx]

**Fig. S1**


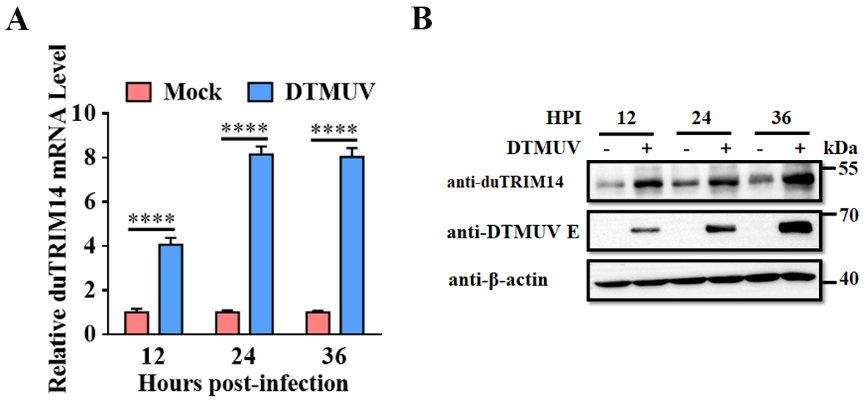


**Fig. S1 TMUV infection induces the expression of duTRIM14 in DEFs.** (A and B) DEFs were inoculated with TMUV at an MOI of 0.1 for the indicated time points. RT-qPCR (A) and western blot (B) were used to measure duTRIM14 protein and mRNA levels. Results from RT-qPCR are presented as means ± SD from three independent experiments. Statistical significance was determined by two-way ANOVA followed by Sidak's multiple comparisons test (*****P* < 0.0001).
