# Supplementary material for: TRIM14 restricts tembusu virus infection through degrading viral NS1 protein and activating type I interferon signaling
Source: PLoS Pathog. 2025 May 28;21(5):e1013200. doi: 10.1371/journal.ppat.1013200 (PMC12118852; doi:10.1371/journal.ppat.1013200)
Supplement: S2 Fig — DEFs were transfected with either control siRNA (siNegative) or siRNA targeting duTRIM14 (siduTRIM14). (A) At 30 h post-transfection, intracellular mRNA was extracted and the abundance of duTRIM14 mRNA was quantified by RT-qPCR. Data are representative of three independent experiments and presented as mean ± SD. Statistical significance was determined by one-way ANOVA followed by Dunett’s multiple comparisons test (***P < 0.001, ****P < 0.0001). (B) Immunoblot analysis of lysates from DEFs transfected with siNegative or siduTRIM14 with an anti-duTRIM14 antibody. (DOCX) [file ppat.1013200.s002.docx]

**Fig. S2**


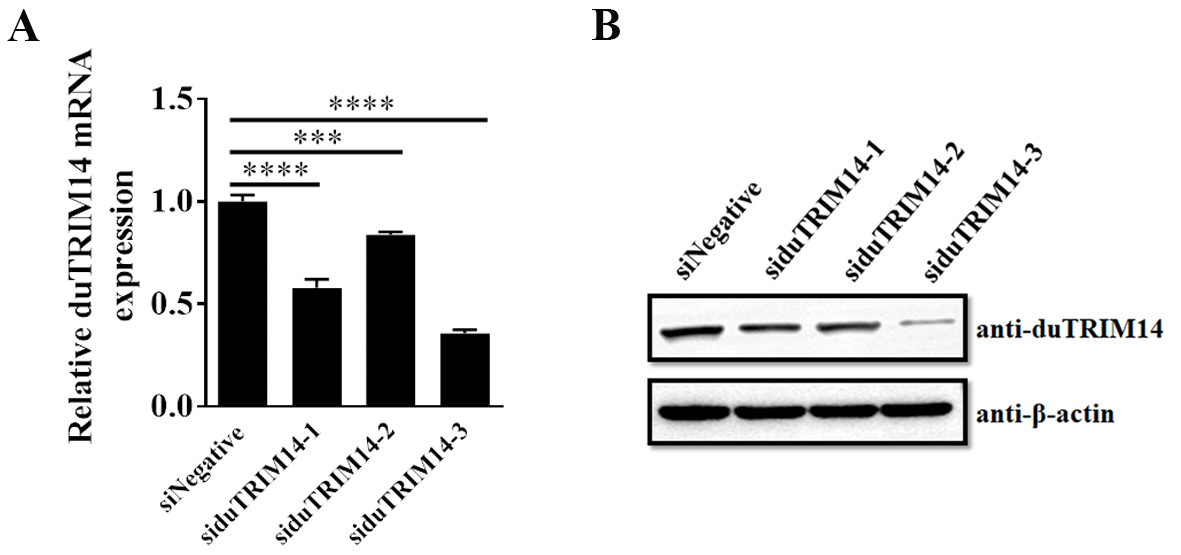


**Fig. S2 Gene silencing efficiency of siRNA targeting duTRIM14 in DEFs.** DEFs were transfected with either control siRNA (siNegative) or siRNA targeting duTRIM14 (siduTRIM14). (A) At 30 h post-transfection, intracellular mRNA was extracted and the abundance of duTRIM14 mRNA was quantified by RT-qPCR. Data are representative of three independent experiments and presented as mean ± SD. Statistical significance was determined by one-way ANOVA followed by Dunett's multiple comparisons test (****P* < 0.001, *****P* < 0.0001). (B) Immunoblot analysis of lysates from DEFs transfected with siNegative or siduTRIM14 with an anti-duTRIM14 antibody.
