# Supplementary figures and images for: TRIM14 restricts tembusu virus infection through degrading viral NS1 protein and activating type I interferon signaling
Source: PLoS Pathog. 2025 May 28;21(5):e1013200. doi: 10.1371/journal.ppat.1013200 (PMC12118852; doi:10.1371/journal.ppat.1013200)

**Fig. S3**

**
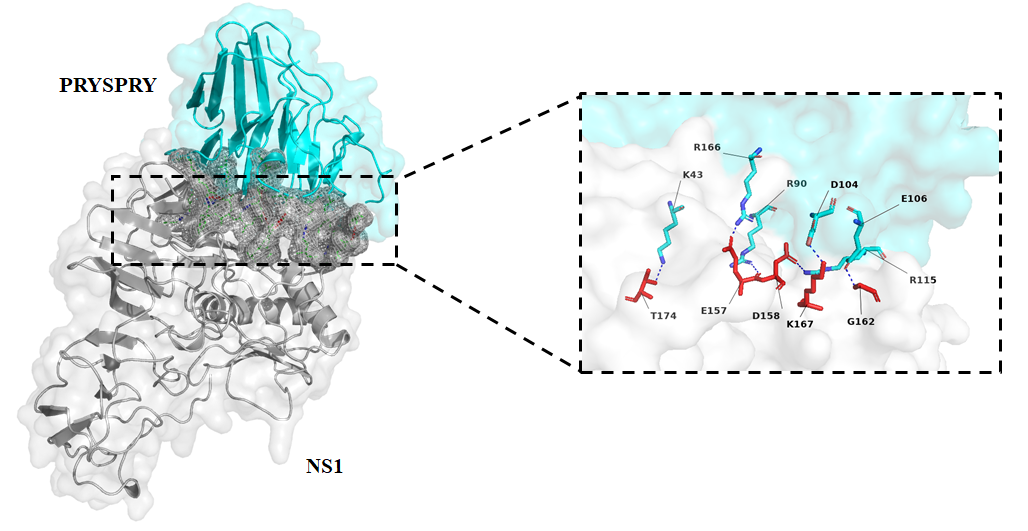
**

**Fig. S3** **Molecular docking prediction results for duTRIM14-PRYSPRY and TMUV NS1 proteins.**

Supplement: S3 Fig — (DOCX) [file ppat.1013200.s003.docx]

**Fig. S4**


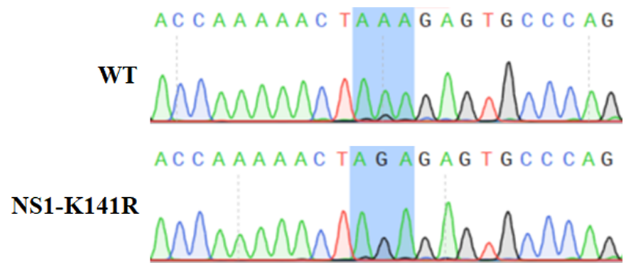


**Fig. S4** **Confirmation of TMUV NS1-K141R mutant virus by Sanger sequencing.**

Supplement: S4 Fig — (DOCX) [file ppat.1013200.s004.docx]
