# Supplementary material for: TRIM14 restricts tembusu virus infection through degrading viral NS1 protein and activating type I interferon signaling
Source: PLoS Pathog. 2025 May 28;21(5):e1013200. doi: 10.1371/journal.ppat.1013200 (PMC12118852; doi:10.1371/journal.ppat.1013200)
Supplement: S5 Fig — The protein sequences of TBK1 between human and duck were compared and analyzed with ESPript 3.0 online software (https://espript.ibcp.fr/ESPript/cgi-bin/ESPript.cgi). (DOCX) [file ppat.1013200.s005.docx]

**Fig.S5**


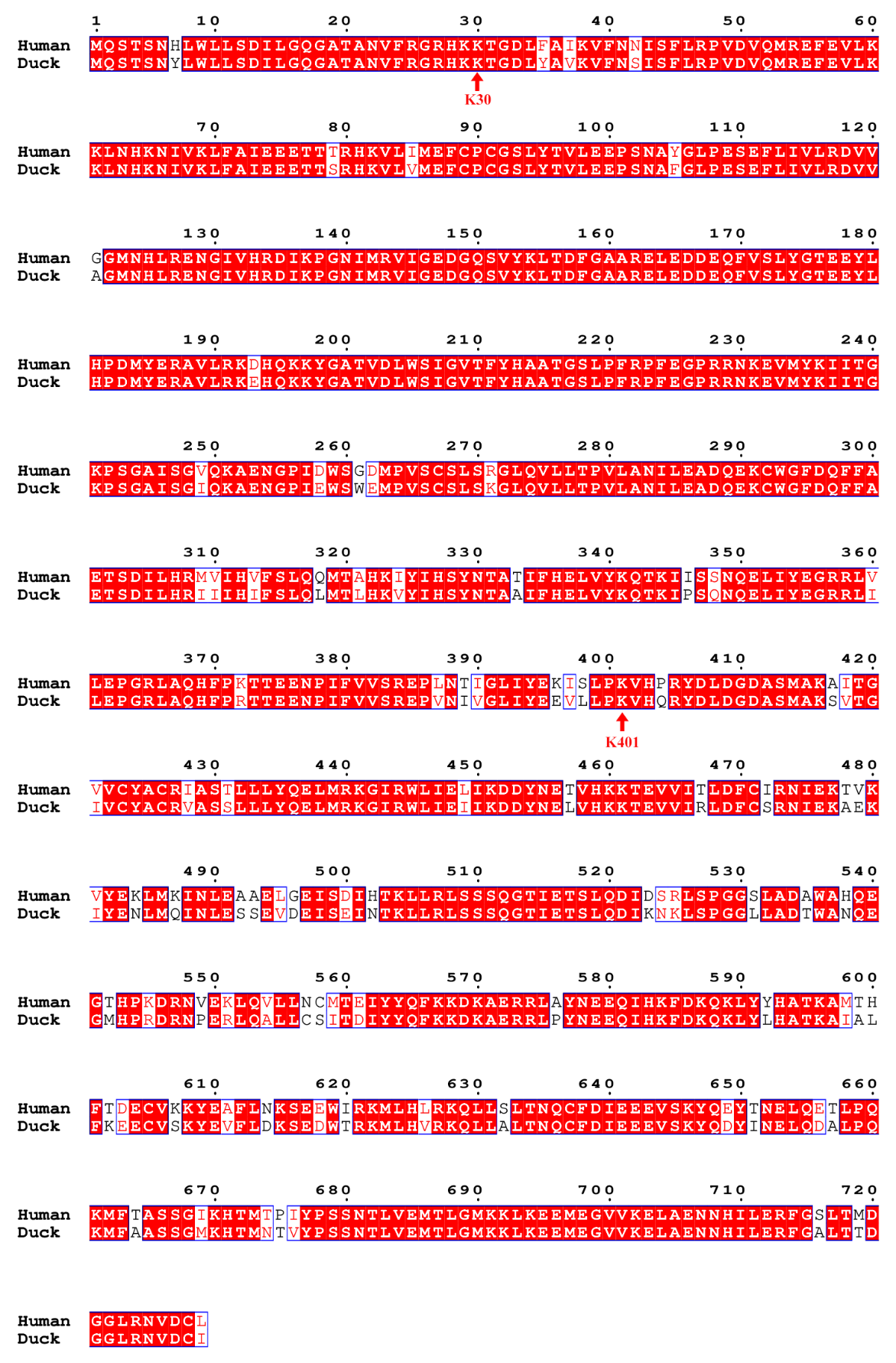


**Fig.S5** **Alignment of TBK1 protein sequences between human and duck.** The protein sequences of TBK1 between human and duck were compared and analyzed with ESPript 3.0 online software (https://espript.ibcp.fr/ESPript/cgi-bin/ESPript.cgi).
