# Supplementary material for: TRIM14 restricts tembusu virus infection through degrading viral NS1 protein and activating type I interferon signaling
Source: PLoS Pathog. 2025 May 28;21(5):e1013200. doi: 10.1371/journal.ppat.1013200 (PMC12118852; doi:10.1371/journal.ppat.1013200)
Supplement: S1 Table — (DOCX) [file ppat.1013200.s006.docx]

**S1 Table.** Primers for RT-qPCR and plasmid construction in this study.

| Primer | Sequence (5’-3’) |
| --- | --- |
| *du**TRIM14-CDS-F* | TATGAATTCATGGCGCTGGGGGAGCCGC |
| *duTRIM14-CDS-R*  *NS1-K139R-F*  *NS1-K139R-R*  *NS1-K141R-F*  *NS1-K141R-R*  *NS1-K189R-F*  *NS1-K189R-R*  *NS1-K205R-F*  *NS1-K205R-R*  *NS1-K347R-F*  *NS1-K347R-R*  *NS1-K349R-F*  *NS1-K349R-R* | TATCTCGAGTTACGTCAGCTTGCATGTGCCAATGGACC  GTCATTGATGGACCAAGAACTAAAGAGTGCCC  AAATGTATTATTTGACATCTTCGGGCTGCTGAAAATAC  ATGGACCAAAAACTAGAGAGTGCCCAGATGAG  CAATGACAAATGTATTATTTGACATCTTCGGGC  GGCACAGCAATTAGAGGAAATAGAGCTG  CATCACTGCGGTGTCACAATCAGTTGTA  TTGGATAGAGAGCAGGAATAATGGAAGCTG  TAGCTCAGGTCACTGTGCACAGCTCTATTTC  GACACGGGGTGCTCAATCGACTTGG  AGCCATGACCTTTGATCTGATCAACATGTCGTC  GACACGGGGTGCTCAATCGACTTGG  AGCCATGACCCTTGATTTGATCAACATGTCGTC |
| *qduTRIM14-F* | CTGAACGCCACCACAAAGAA |
| *qduTRIM14-R* | GGTCGTCAGACAACTTCAGC |
| *qDTMUV-NS1-F* | CCATCCACACCAAGGAGACT |
| *qDTMUV-NS1-R* | TGCTTACCCACATGTTGTGC |
| *qDTMUV-E-F* | ACCATGGACAGGGTCATCAG |
| *qDTMUV-E-R* | GGAGGGCTCCTTCTTGTGAT |
| *qdu*IFNβ*-F* | ACATCCTTTTGGACACCGACAA |
| *qdu*IFNβ*-R* | TTGGACTGCTGAGGATGTTGA |
| *qduViperin-F* | GCCGAGAGTATGCTGTTGCTT |
| *qduViperin-R* | AATGAGCAGGCACTGGAACAC |
| *qduPKR-F* | GTGAAGGTGGTTTTGGGAAT |
| *qduPKR-R* | TCACGCTTCACCTTCTCCTTA |
| *qduZAP-F* | GCTCCTCTTCATTGCTTCGA |
| *qduZAP-R* | CCACTGGCCTTGGTCATTCT |
| *qduGAPDH-F* | CAAGGCTGAGAATGGGAAACTT |
| *qduGAPDH-R* | GCATCTGCCCACTTGATGTT |
