# Supplementary material for: TRIM14 restricts tembusu virus infection through degrading viral NS1 protein and activating type I interferon signaling
Source: PLoS Pathog. 2025 May 28;21(5):e1013200. doi: 10.1371/journal.ppat.1013200 (PMC12118852; doi:10.1371/journal.ppat.1013200)
Supplement: S2 Table — (DOCX) [file ppat.1013200.s007.docx]

**S2 Table.** The sequences of siRNAs used in the study.

| siRNA | Sequences (5' to 3') |
| --- | --- |
| siduTRIM14-1  siduTRIM14-2  siduTRIM14-3  siNegative control | 5' GCCUGGCGAGAAAUUUAAUTT 3'  5' AUUAAAUUUCUCGCCAGGCTT 3'  5' GCAUCUCAUGACAGGGCUUTT 3'  5' AAGCCCUGUCAUGAGAUGCTT 3'  5' GGAAUACGACAGCCUUCAUTT 3'  5' AUGAAGGCUGUCGUAUUCCTT 3'  5' UUCUCCGAACGUGUCACGUTT 3'  5' ACGUGACACGUUCGGAGAATT 3' |
